# The Financial and Operational Effects of COVID-19 on Ohio’s Long-Term Care Residential Settings

**DOI:** 10.1093/geroni/igab046.3445

**Published:** 2021-12-17

**Authors:** Katherine Kennedy, Robert Applebaum, Kathryn Brod

**Affiliations:** 1 LTSS COIN Providence VA Medical Center; 2 Miami University, Oxford, Ohio, United States; 3 LeadingAge Ohio, Columbus, Ohio, United States

## Abstract

The COVID-19 pandemic has had a drastic impact on Ohio’s long-term care facilities. Yet, months into the crisis, the financial ramifications and workforce shortage were unknown. In partnership with the Scripps Gerontology Center at Miami University, LeadingAge Ohio and the Ohio Health Care Association developed an online survey that was launched in July 2021. Response rates were 46.4% for skilled nursing facilities (SNFs; N=446) and 35.8% for residential care facilities (RCFs; N=287). Core questions compared the first quarters of 2020 and 2021. Declines in operating revenues (-11.7% SNFs; -10% RCFs) and rising labor costs per patient day (17.9% SNFs; 16.1% RCFs) contributed to most providers experiencing a financial loss in the most recent month (78% SNFs; 66% RCFs). The increased documented use of agency staff is an important finding of this work; 62% of SNFs and 34% of RCFs spent money on agency staff. Despite increases in starting wages, the labor crisis remains severe. As of July 2021, SNFs had an average of 19.51 open positions, of which 9.82 were for state-tested nurse aides and 5.65 were for nurses. RCFs had an average of 8.83 open positions, of which 4.24 were for resident care assistants and 1.89 were for nurses. The challenges faced by the long-term care industry have rightly focused on the deleterious impacts of COVID on residents and staff. But these data also suggest that the financial impacts on the industry are serious and will likely shape access and provision of care in the future.

